# Root-associated fungal microbiota of the perennial sweet sorghum cultivar under field growth

**DOI:** 10.3389/fmicb.2022.1026339

**Published:** 2022-10-26

**Authors:** Gui-Hua Lu, Kezhi Zheng, Rui Cao, Aliya Fazal, Zhiye Na, Yuanyuan Wang, Yonghua Yang, Bo Sun, Hongjun Yang, Zhong-Yuan Na, Xiangxiang Zhao

**Affiliations:** ^1^Jiangsu Key Laboratory for Eco-Agricultural Biotechnology Around Hongze Lake, School of Life Sciences, Huaiyin Normal University, Huai’an, China; ^2^State Key Laboratory of Pharmaceutical Biotechnology, School of Life Sciences, Nanjing University, Nanjing, China; ^3^Yunnan Eco-Agriculture Research Institute, Kunming, China

**Keywords:** *Sorghum bicolor* (L.), perennial sweet sorghum cultivar, rhizosphere, root, high-throughput sequencing, fungal rRNA gene internal transcribed spacer 1, indicators of sorghum yield and protein content

## Abstract

Root-associated fungal microbiota, which inhabit the rhizosphere, rhizoplane and endosphere, have a profound impact on plant growth and development. *Sorghum bicolor* (L.) Moench, also called broomcorn or sweet sorghum, is a multipurpose crop. The comparison between annual and perennial sweet sorghum cultivars in terms of plant growth, as well as their interactions with belowground fungal microbiota, is still poorly understood, although there has been growing interest in the mutualism between annual sweet sorghum and soil bacteria or bacterial endophytes. In this study, the perennial sweet sorghum cultivar N778 (N778 simply) and its control lines TP213 and TP60 were designed to grow under natural field conditions. Bulk soil, rhizosphere soil and sorghum roots were collected at the blooming and maturity stages, and then the fungal microbiota of those samples were characterized by high-throughput sequencing of the fungal ITS1 amplicon. Our results revealed that the alpha diversity of the fungal microbiota in rhizosphere soil and root samples was significantly different between N778 and the two control lines TP213 and TP60 at the blooming or maturity stage. Moreover, beta diversity in rhizosphere soil of N778 was distinct from those of TP213 and TP60, while beta diversity in root samples of N778 was distinct from those of TP213 but not TP60 by PCoA based on Bray–Curtis and WUF distance metrics. Furthermore, linear discriminant analysis (LDA) and multiple group comparisons revealed that OTU4372, a completely unclassified taxon but with symbiotroph mode, was enriched in sorghum roots, especially in N778 aerial roots at the blooming stage. Our results indicate that *Cladosporium* and *Alternaria*, two fungal genera in the rhizosphere soil, may also be dominant indicators of sorghum yield and protein content in addition to *Fusarium* at the maturity stage and imply that the perennial sweet sorghum N778 can primarily recruit dominant psychrotolerant bacterial taxa but not dominant cold-tolerant fungal taxa into its rhizosphere to support its survival below the freezing point.

## Introduction

The rhizosphere is a hotspot of plant–microbe interactions and home to a rich microbiota, including bacteria, fungi, and archaea (Reinhold-Hurek et al., 2015). It allows microbes to enter the root interior as endophytes (Liu et al., 2017). Endophytes are nonpathogenic microbes – often bacteria or fungi – that reside within host plants (Philippot et al., 2013). They are ubiquitous in nature and frequently engage in mutualistic, antagonistic, and sporadically parasitic relationships with their hosts (Gouda et al., 2016). The host plants, in turn, are found to influence associated microbial communities, indicating a dynamic link between the root and soil microbiome (Bai et al., 2022). For instance, Bergelson et al. recently identified *Arabidopsis* plant genetic traits as the primary drivers of bacterial or fungal community recruitment. Other studies have reported that plant genotypic diversity influences fungal composition and functions (Gehring et al., 2017).

Fungal endophytes impact plant growth and development by enhancing nutrient uptake, pathogen suppression, phytohormone production, and stress tolerance (Rodriguez et al., 2008; Bamisile et al., 2018; Jaber, 2018; Rana et al., 2020). Behie et al. (2012) was the first to report the entomopathogenic fungus *Metarhizium robertsii* invading bean plants (*Phaseolus vulgaris*) and switchgrass (*Panicum virgatum*) and directly translocating nitrogen from insects to plants (Behie et al., 2012). Endophytic fungi of the genus *Daldinia*, which contain fungicidal compounds, have been shown to inhibit the growth of the plant pathogen *Colletotrichum acutum* (Khruengsai et al., 2021). Previous studies reported a number of endophytic fungal species capable of producing indole-3-acetic acid (IAA) and gibberellins (GAs), thereby exerting a number of beneficial effects on shoot and root development (Khan et al., 2012; Waqas et al., 2012). Similarly, the GA-producing endophytic fungus *Cladosporium sphaerospermum* was reported to stimulate the growth of rice and soybean plants (Hamayun et al., 2009; Waqas et al., 2014). In addition, *Glomus mosseae*-inoculated rice plants showed increased tolerance to low temperature (15°C), with improved phosphorus uptake, enzymatic activity, and elevated nitric oxide plus jasmonic acid concentrations (Liu et al., 2013). In addition, the diversity and structure of both rhizospheric and endophytic soil fungal communities are essential for plant health and soil fertility, while an imbalance can cause a rapid accumulation of fungal pathogens, hence lowering agricultural yields (Miao et al., 2016; Zhu et al., 2020).

Among all fungal associations, mycorrhizal associations play a pivotal role both within the rhizosphere and through the establishment of mutually beneficial endo/ecto-mycorrhizal relationships (Harrison, 2005; Genre et al., 2020). Arbuscular mycorrhizal fungi (AMF), which belong to the phylum Glomeromycota, are by far the most prevalent endomycorrhiza, being associated with more than 78% of land vascular plants by providing enhanced phosphorus, nitrogen and other mineral nutrient access and protection from drought, salt, heavy metal and pathogen stresses (Latef et al., 2016; Tedersoo et al., 2020).

*Sorghum bicolor* (L.) Moench, also called sweet sorghum, is a multipurpose crop belonging to the family Poaceae (Mareque et al., 2018). Although there has been growing interest in the mutualism between soil bacteria and annual sweet sorghum and bacterial endophytes associated with sorghum have been found to improve sorghum growth and development (Mareque et al., 2018; Heijo et al., 2021; Lopes et al., 2021; Sun et al., 2021), the comparison between annual and perennial sweet sorghum cultivars in terms of plant growth, as well as their interactions with belowground fungal microbiota, is still poorly understood.

Our recent study revealed not only notable differences in plant traits between the perennial sweet sorghum cultivar N778 and the annual counterpart but also the significant enrichment of *Pseudarthrobacter* and *Pseudomonas* in the rhizosphere soil of N778 (Lu et al., 2022). However, to identify the key microbes that assist the perennial sweet sorghum in being cold tolerant and exhibiting notable plant traits, a thorough analysis encompassing both bacterial and fungal species is needed. We therefore intend to compare the fungal microbiota associated with the roots (rhizosphere, rhizoplane, and endosphere) of both the perennial sweet sorghum and the annual sorghum to obtain insights into the functional traits of key microbes. At two different growth stages, fungal communities in all three root-associated compartments were analyzed and compared.

## Materials and methods

### Plant materials

NaPBS778 (N778 simply), the perennial sweet sorghum cultivar., was depicted in our prior study (Lu et al., 2022). TP60 and TP213 were used as control sorghum lines, which were two variants of the F11 recombinant inbred line populations generated through cross fertilization of T70 × P607 sorghum cultivars (Dong et al., 2013).

### Field design, management and soil type

The design, management, and soil type of the experimental field on the Huaiyin Normal University (HNU) campus for N778 and the control lines TP60 and TP213 were mentioned in our prior study (Lu et al., 2022).

### Sampling and harvesting

Plants of different sorghum genotypes were dug out, and then bulk soil, rhizosphere soil and root samples from different compartments at the blooming and maturity stages were collected cautiously as mentioned before (Lu et al., 2018) with some adjustments (Lu et al., 2022). Bulk soil, roots together with rhizosphere soil of the control line TP213 were sampled on Oct. 11, 2020. Roots of the perennial sweet sorghum cultivar N778 and two control lines at the maturity stage were collected on December 11 or 12, 2020, under cold temperatures with a daily range from −1°C to 10°C for more than a week. In fact, sorghum plants suffered below freezing temperatures from December 3 to 5, 2020, before sampling according to the historical climate information in December, 2020. After most rhizosphere soils were sampled by brushing with clean tooth brushes, sorghum primary or aerial roots were scissored into approximately 5 cm long segments, vigorously shaken, and thoroughly washed at least three times in sterile 50 ml tubes with pH 7.3 phosphate-buffered saline (PBS) until the supernatant became clear and no pellet remained in the bottom of the tubes after root segments with PBS were centrifugated at 10,000 ×  *g* for 2 min at 4°C. Furthermore, sterile 50 ml tubes containing clean root segments were centrifugated again at 10,000 × g for 2 min at 4°C, and a micropipette was used to remove the tiny PBS remaining at the bottom of the tubes. Finally, centrifugally dried and clean root segments of sorghum were stored at −65°C before being pulverized in liquid nitrogen with a mortar and pestle for extracting metagenomic DNA.

### Metagenomic DNA extraction from root and soil samples

In liquid nitrogen, frozen root segments were pulverized into extremely fine powders before DNA extraction. Then, total metagenomic DNA was extracted from approximately 0.30 g per sample using a DNeasy PowerSoil Kit (Qiagen, CA, United States) according to the instructions of the manufacturer with some adjustments, in which FastPrep-24 (MP Biomedicals, CA, United States) was used to homogenize the soil and root samples. All DNA samples were then stored at −65°C before the DNA integrity was tested using 1% agarose gel electrophoresis.

### ITS1 amplicon high-throughput sequencing *via* Illumina MiSeq

The fusion primers containing the P5 or P7 Illumina adapter, an 8 nucleotide (nt) barcode, and the gene-specific primer pair 1737F (5’-GGAAGTAAAAGTCGTAACAAGG-3′) and 2043R (5’-GCTGCGTTCTTCATCGATGC-3′; Zhang et al., 2016) were used to amplify the fungal ITS1 region. After triple PCR amplification, purification of the PCR products, library quality assessment, and quantification of the libraries, paired-end 300 bp high-throughput sequencing was performed on the Illumina MiSeq platform (Illumina, CA, USA) as mentioned by Zhang et al. (2016). We deposited the raw data from 79 samples into the China National Gene Bank Database (CNGBdb^1^) and released it on September 18, 2022. The CNGB Sequence Archive (CNSA) accession number of raw data from 79 samples is CNP0003362.^2^

### Analysis of amplicon data

Analysis of ITS1 amplicon data was performed as previously reported by Sun et al. (Sun et al., 2021) with some modification. Briefly, raw reads were initially filtered to remove those with average base quality scores under 20, ambiguous N bases, and lengths shorter than 50 nt through Fastp (v0.19.6; Chen et al., 2018). In the next step, clean tags were produced by combining the paired-end reads with high-quality through FLASH (v1.2.11; Magoc and Salzberg, 2011) and removing singletons and the chimeric sequence. Then, UPARSE (v7.0.1090; Edgar, 2013) was used to generate operational taxonomic units (OTUs) with 97% sequence similarity from effective tags. Additionally, the resulting representative OTUs were classified taxonomically utilizing the Ribosomal Database Project (RDP) classifier (v2.11; Wang et al., 2007) based on the Unite database (v8.0^3^; Nilsson et al., 2019). The online Majorbio Cloud Platform^4^ was used to further analyze amplicon data.

### Analysis of fungal potential function by FUNGuild

The fungal potential function value of each OTU was analyzed by FUNGuild, and a table of the fungal potential function of each OTU with its value was generated. Then, a composition barplot of different groups was made on the online Marjorbio Cloud platform. The trophic mode includes pathotrophs, symbiotrophs, and saprotrophs. The term “guild” refers to a group of some species that are classified into the same type by adopting similar methods in the absorption and utilization of environmental resources, regardless of whether they are phylogenetically related. There are 12 guilds, including animal pathogens, foliar endophytes, ectomycorrhizal fungi, arbuscular mycorrhizal fungi, ericoid mycorrhizal fungi, lichen parasite fungi, lichenized fungi, mycoparasites, undefined root endophytes, plant pathogens, undefined saprotrophs and wood saprotrophs.

### Statistical analysis

Welch’s *t*-test (for unknown variance) or the Wilcoxon rank-sum test was performed for pairwise group comparisons. Multiple group comparisons were conducted utilizing the Kruskal–Wallis H test followed by a *post hoc* Tukey–Kramer test through the online Majorbio Cloud Platform.^5^ The software R (v3.3.1) vegan package was used to perform analysis of similarities (ANOSIM) and Adonis (permutational multivariate analysis of variance) based on weighted UniFrac (WUF) or Bray–Curtis distance metrics.

## Results

### Overall fungal ITS1 amplicon sequencing data

A total of 5,260,644 clean tags with an average number of 66,590 per sample were generated from 79 samples by fungal ITS1 (1737F to 2043R) amplicon high-throughput sequencing through the Illumina MiSeq platform (Supplementary Table S1). The mean length of clean tags per sample ranged from 213.78 to 270.92 nt, and the Q30 average value of clean tags per sample ranged from 98.3487 to 99.5860% (Supplementary Table S1). Approximately 65,965 effective taxonomic tags at 97% sequence similarity were assigned to each sample (Supplementary Table S1). Additionally, the total number of OTUs before and after normalization was 5,909 and 5,785, respectively, belonging to 15 phyla and 56 classes (Supplementary Tables S2, S3). In addition, the average numbers of OTUs per sample before and after normalization were 749.8 ± 449.47 and 665.7 ± 394.14, respectively (Supplementary Table S1).

### Alpha diversity of fungal microbiota in different sorghum compartments

All 79 samples were normalized based on the minimal number of 43,529 effective taxonomic tags generated from the sample TP_2MARt (Supplementary Tables S2, S3) before beta diversity analysis and nine common index data points for alpha diversity (Supplementary Table S4).

**Figure 1:**
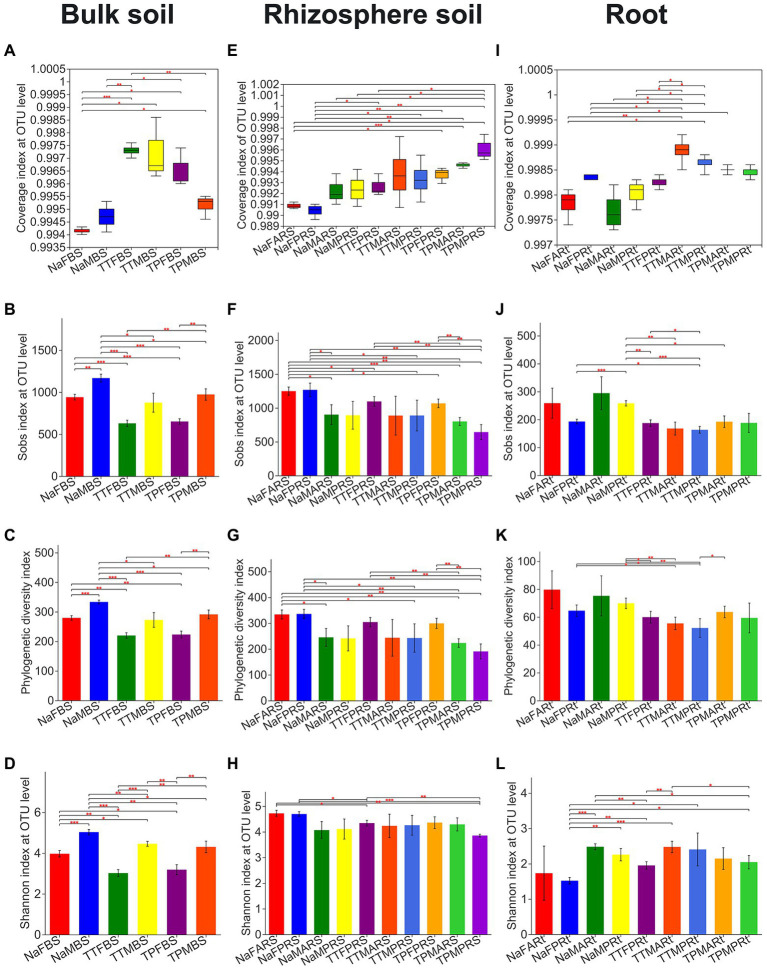
Observed OTU richness (Sobs), phylogenetic diversity, Shannon evenness and Good’s coverage (Coverage) indices for fungal microbiota across different compartments of 3 sorghum lines. **A**-**D**, **E**-**H**, and **I**-**L** reprent Coverage, Sobs, PD, and Shannon indices of bulk soil, rhizosphere soil, and root compartents,respectively. Statistical differences between pairwise groups were determined by using Welch’s *t*-test. *,** and *** indicate *p* < 0.05, *p* < 0.01 and *p* < 0.001, respectively. For the group name, Na, TT and TP refer to the perennial sweet sorghum NaPBS778 and the control lines TP213 and TP60, respectively; F and M refer to the blooming stage and the maturity stage, respectively; A and P represent the latest aerial roots and the primary roots, respectively; BS, RS, and Rt represent bulk soil, rhizospheric soil and roots, respectively.

The Good’s coverage (coverage) index of all 25 groups exceeded 98.9% (Supplementary Table S4), although there were significant or very significant differences (Welch’s *t*-test) among the same compartment between different genotypes (Figures 1A,E,I), and the rarefaction curve of coverage, along with the other three indices, was almost at saturation (Supplementary Figure S1), demonstrating that the sequencing depth was sufficient to capture the diversity of fungal endophyte communities as well as enough detectable species. Additionally, no significant differences were found in coverage and phylogenetic diversity in the overall distribution between most pairwise groups except the rhizosphere soil and root samples of TP213 at the maturity stage (TTMARS, TTMPRS, TTMARt, and TTMPRt) by the Wilcoxon rank-sum test (Supplementary Figures S2A,C). For Sobs, there were significant differences in the overall distribution between the maturity primary root samples of TP213 (TTMPRt) and almost all other groups (Supplementary Figure S2B).

In the bulk soil compartment, the observed OTU richness (Sobs), phylogenetic diversity (PD), and Shannon evenness indices of the bulk soil of N778, whose group name began with Na, at either the blooming or maturity stage were significantly or very significantly higher than those of TP213 and TP60, whose group name began with TT and TP, respectively (Figures 1B–D). Additionally, the Sobs, PD, and Shannon index of the sample groups at the maturity stage were considerably or remarkably higher than those at the blooming stage (Figures 1B–D).

In the rhizosphere compartment, the Sobs index of rhizospheric soil of N778 aerial roots at the blooming stage (NaFARS) was significantly higher than those of TP213 and TP60 at the blooming stage as well as at the maturity stage (Figure 1F). Furthermore, the Sobs and PD indices of rhizosphere soil of N778 primary roots at the blooming stage (NaFPRS) were considerably higher than those of rhizosphere soil groups at the maturity stage (Figures 1F,G). The Shannon index of rhizosphere soil of N778 aerial roots at the blooming stage (NaFARS) and N778 primary roots at the blooming stage (NaFPRS) was significantly higher than that of TP213 primary roots at the blooming stage (TTFPRS) and TP60 primary roots at the maturity stage (TPMPRS; Figure 1H).

In the root compartment, N778 primary roots at the maturity stage (NaMPRt) had a considerably higher Sobs index than TP213 and TP60 root samples, as well as N778 primary roots at the blooming stage (NaFPRt; Figure 1J). The PD index of N778 primary roots at the blooming and maturity stages was significantly higher than that of TP213 root samples (Figure 1K). The Shannon index of N778 aerial roots at the maturity stage (NaMARt) was significantly higher than that of TP213 primary roots at the blooming stage (TTFPRt) and TP60 primary roots at the maturity stage (TPMPRt), while the Shannon index of N778 primary roots at the blooming stage (NaFPRt) was significantly or very significantly lower than that of many other root groups (Figure 1L).

### Beta diversity of fungal microbiota in different sorghum compartments

**Figure 2:**
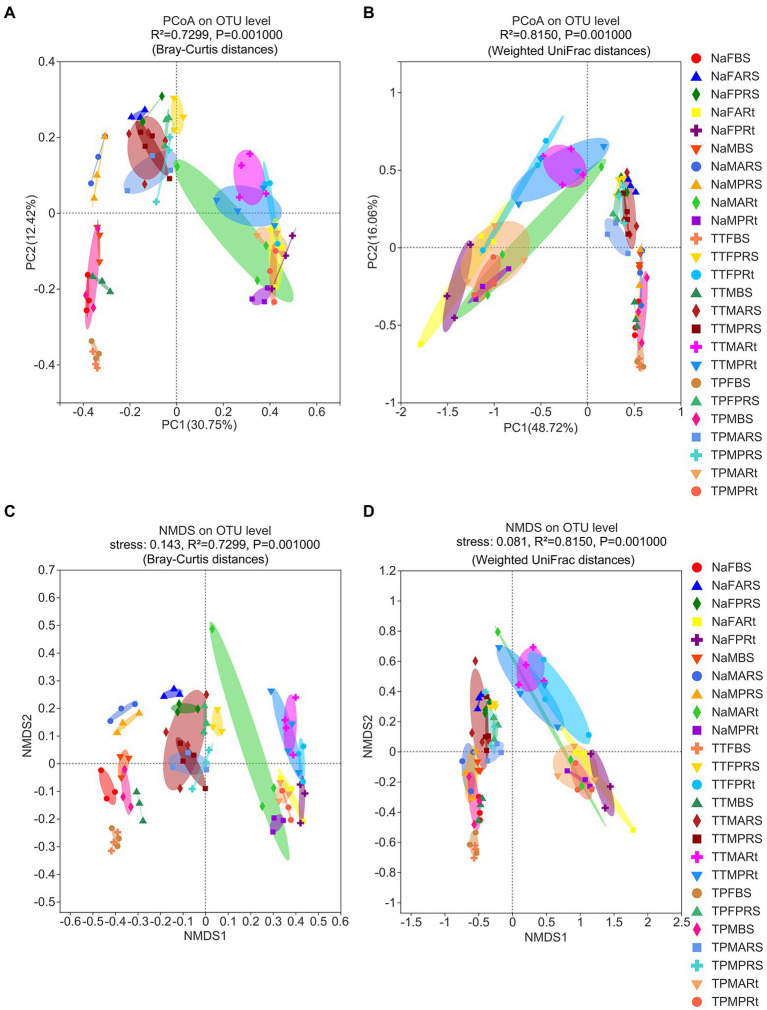
Beta diversity of fungal microbiota in different compartments of three sorghum lines at the OTU level analyzed by nonmetric multidimensional scaling (NMDS) and principal coordinate analysis (PCoA) with statistical results from Adonis. **(A)** PCoA based on Bray–Curtis distance, and the statistical results from ANOSIM are shown as *R* = 0.8070 and *p* = 0.001. **(B)** PCoA based on weighted UniFrac (WUF) distance, and the statistical results from ANOSIM are shown as *R* = 0.7473 and *p* = 0.001. **(C)** NMDS based on Bray–Curtis distance metrics; the stress value was 0.143. **(D)** NMDS based on WUF distance metrics, and the stress value was 0.081. The treatment details are shown in Figure 1.

The variations in beta diversity in the overall samples at the OTU level were initially investigated using principal coordinate analysis (PCoA). PCoA results based on Bray–Curtis distance metrics indicated that there were significant differences across bulk soil, rhizosphere soil, and root samples (Figure 2A, see also Figure S3A), except that one root sample was distributed on the top left side of Figure 2A (see also Supplementary Figure S3A). However, the fungal microbiota in bulk soil samples did not differ significantly from those in rhizospheric soil samples in accordance with the PCoA results based on WUF distance metrics (Figure 2B, see also Supplementary Figure S3B).

Next, similarity of the fungal microbiota composition of different compartments was carried out by nonmetric multidimensional scaling (NMDS) analysis. NMDS based on Bray–Curtis or WUF distance metrics, followed by ANOSIM and Adonis statistical analysis, uncovered similar results by PCoA (Figures 2C,D). Additionally, both stress values were less than 0.2 (Figures 2C,D), indicating that the NMDS analysis results were valid.

Then, fungal microbiota in bulk soil, rhizosphere soil and root compartments were analyzed separately to identify the difference between the perennial sweet sorghum N778 and two control lines TP213 and TP60. PCoA results based on WUF and Bray–Curtis distance metrics revealed that fungal microbiota in bulk soil of N778 at the maturity stage were clearly distinct from not only those of TP213 and TP60 at the blooming or maturity stage but also those of N778 at the blooming stage, whereas no significant difference existed between TP213 and TP60 bulk soil at the blooming or maturity stages (Supplementary Figures S4A,B).

Similar situations were found in the rhizosphere soil compartment (Supplementary Figures S4C,D), while no significant differences were found between the rhizosphere soil of aerial roots and that of primary roots (Supplementary Figures S4C,D). With regard to the root compartment, fungal microbiota in N778 aerial or primary root samples appeared to be separated from those of TP213 root samples but not separated from those of TP60 root samples (Supplementary Figures S4E,F).

**Figure 3:**
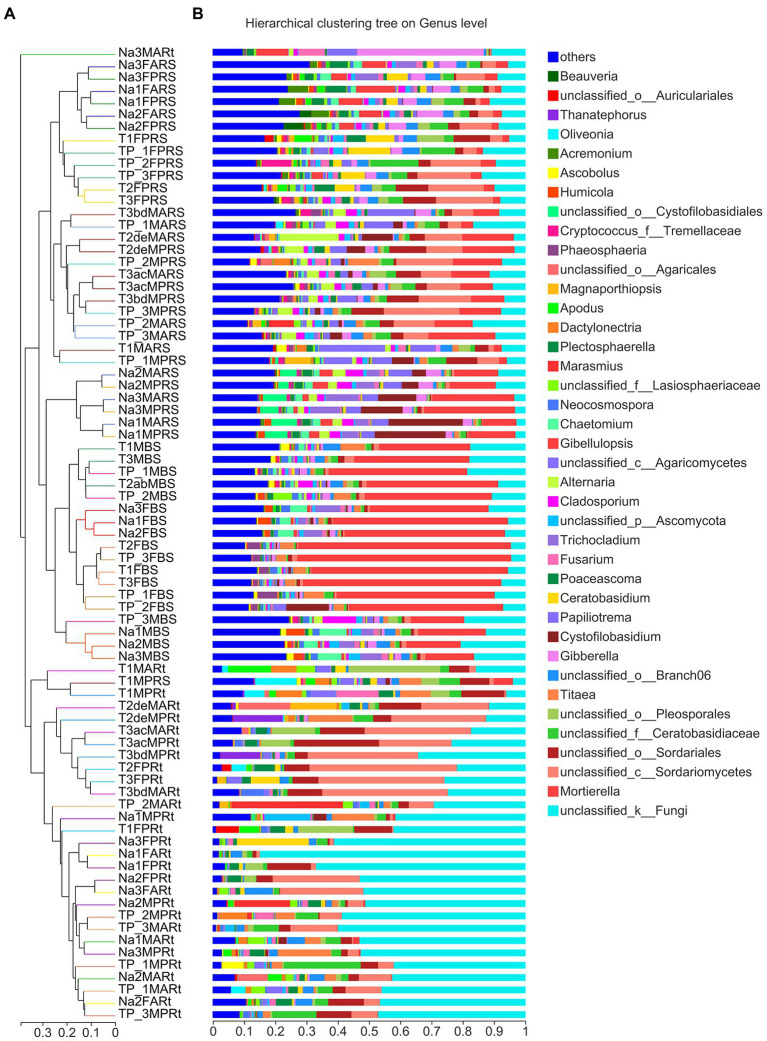
Hierarchical clustering of all samples according to Bray–Curtis distance and fungal microbiota composition of each sample at the genus level. **(A)** Hierarchical clustering tree of all 79 samples belonging to 25 groups; the branch length represents the distance between samples. **(B)** The fungal composition of 79 samples at the genus level displayed *via* stacked column charts. Each sample’s dominant genera are displayed in the stacked column, and those genera with relative abundances less than 5% were combined as others. The treatment details are shown in Figure 1, in addition to sample names starting with uppercase letter T and digits referring to the sorghum line TP213.

Furthermore, based on Bray–Curtis distance metrics, a hierarchical clustering tree was constructed to detect the differences at the genus level in bulk soil, rhizospheric soil and samples among N778, TP213 and TP60 (Figure 3). First, almost all root samples except Na3MARt were clearly clustered into a main clade; moreover, almost all root samples of TP213, except for T1FPRt, were clustered into a subclade (Figure 3A). Next, all bulk soil and rhizospheric soil samples except T1MPRS were clustered into the other main clade; however, it was strange that six rhizospheric soil samples of sweet sorghum N778 at the maturity stage were not only clustered into a subclade but also clustered together with all bulk soil samples (Figure 3A). Then, all the other rhizospheric samples were clustered together; six rhizospheric soil samples of N778 at the blooming stage were clustered together into a subclade (Figure 3A).

### Fungal microbiota composition in different sorghum compartments

**Figure 4:**
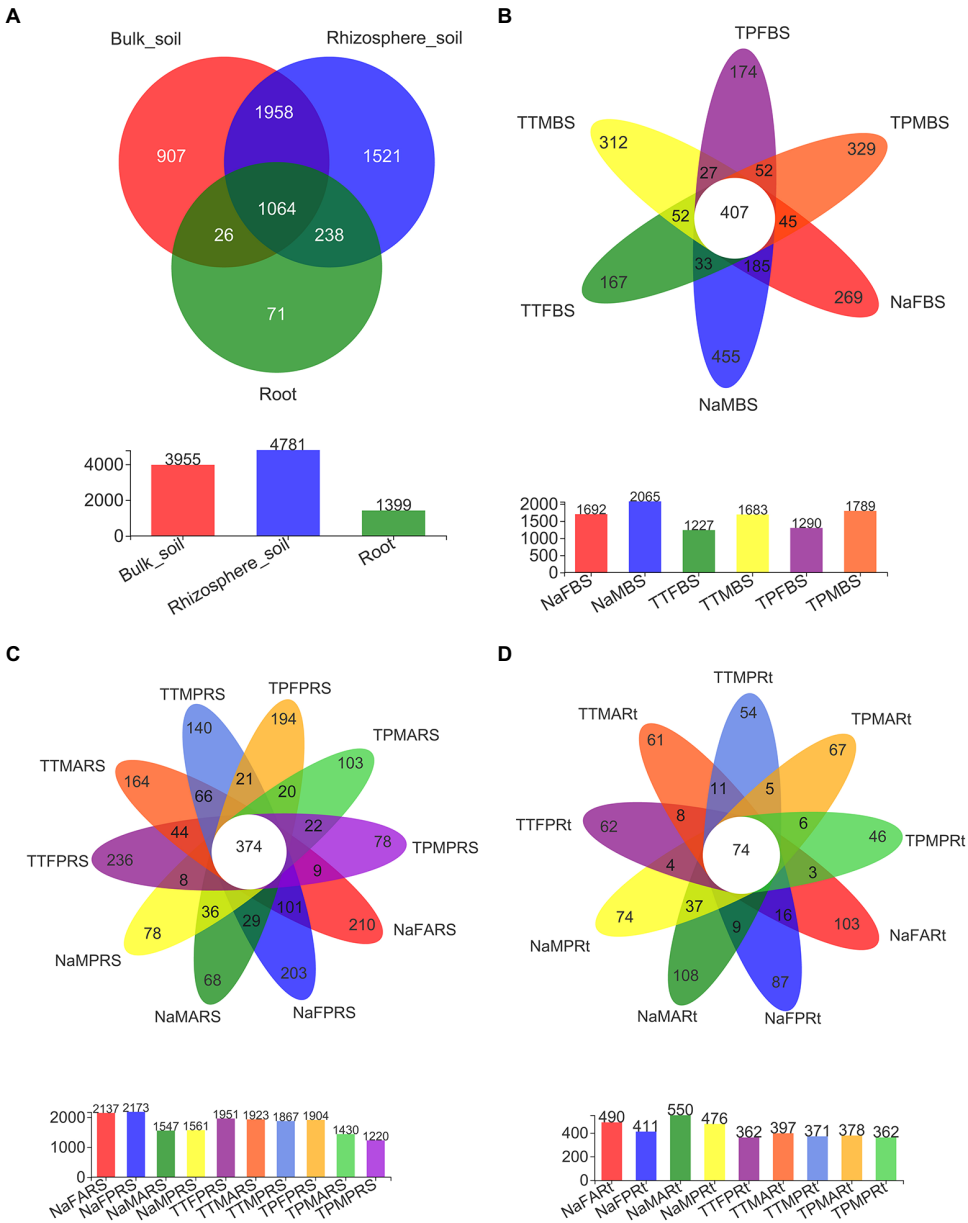
Shared and unique OTUs in different compartments and in different groups of N778 and two control sorghum lines. **(A)** Shared and unique OTUs in bulk soil, rhizosphere soil and root compartments. Shared and unique OTUs in different groups of bulk soil **(B)**, rhizosphere soil **(C)** and roots **(D)** between the 3 sorghum lines. The treatment details are shown in Figure 1.

Among a total of 5,785 OTUs generated from all 79 samples after normalization (Supplementary Table S3), 1,064 OTUs were shared across all three compartments, while 907, 1521, and 71 OTUs were unique to the bulk soil, rhizosphere and root compartments, respectively (Figure 4A). There were 407 OTUs shared across six bulk soil groups among the total of 3,955 OTUs in bulk soil compartments (Figure 4B), while there were 374 OTUs shared across 10 rhizosphere soil groups among the total of 4,781 OTUs in rhizosphere compartments (Figure 4C). Only 74 OTUs were shared across 9 root groups among the total of 1,399 OTUs in root compartments, while 87, 103, 74 and 108 OTUs were found only in the primary roots, aerial roots of sweet sorghum N778 at the blooming stage, and the primary roots and aerial roots of sweet sorghum N778 at the maturity stage, respectively (Figure 4D). Furthermore, the unique number of OTUs in N778 root samples was greater than those in TP213 or TP60 root samples (Figure 4D). Additionally, the top ten OTUs were OTU4372, OTU3906, OTU3596, OTU3754, OTU3672, OTU4147, OTU4122, OTU4168, OTU4022, and OTU3719 among a total of 5,785 OTUs after normalization (Supplementary Table S3), and the top ten OTUs had relative abundances over 44% (Supplementary Table S3). Among the 710 genera, six genera of the top ten were unclassified, and four were known (i.e., *Mortierella*, *Titaea*, *Gibberella*, and *Cystofilobasidium*; Figure 3B; Supplementary Figure S5). The top 1^st^ genus was not only an unclassified genus, family, order, and class of fungi but also an unclassified phylum of fungi, which accounted for 20.168% of all 79 samples (Supplementary Table S5, sheet of genus_full). The other five unclassified genera belonged to the class Sordariomycetes, the order Sordariales, the family Ceratobasidiaceae, the order Branch06, and the order Pleosporales (Figure 3B; Supplementary Figure S5).

**Figure 5:**
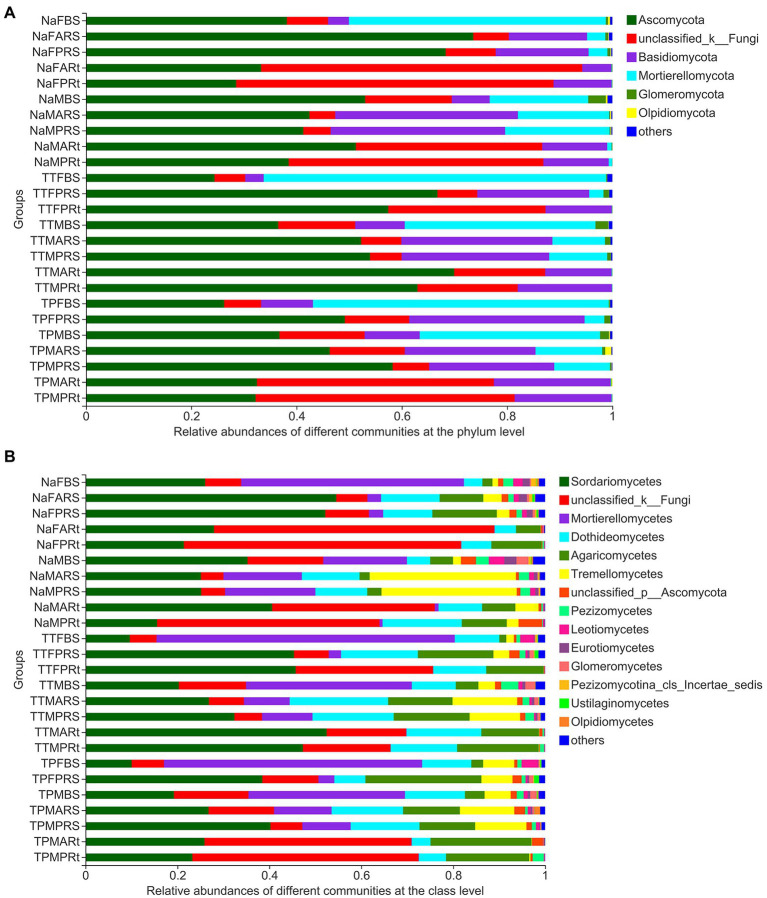
Composition of 25 groups at the phylum **(A)** and class **(B)** levels. Taxa with rich abundances are displayed on the stacked column, and those taxa with relative abundances less than 1% were combined as others. The treatment details are shown in Figure 1.

The top six phyla in all 25 groups, including all 79 samples, were Ascomycota, an unclassified phylum, Basidiomycota, Mortierallomycota, Glomeromycota, and Olpidiomycota (Figure 5A), while other phyla with relative abundances of less than 1% were combined as others.

At the class level, the top nine fungal classes, including two unclassified classes, Sordariomycetes, Mortierellomycetes, Dothideomycetes, Agaricomycetes, Tremellomycetes, Pezizomycetes, and Leotiomycetes, among the top 15 classes, were also dominant taxa and accounted for most relative abundances in all 25 groups (Figure 5B).

### Fungal taxa enriched in rhizosphere and root compartments of the perennial sweet sorghum cultivar N778

First, linear discriminant analysis (LDA) by LEfSe based on all (samples)-against-all (samples) was used to find the fungal taxa significantly enriched in rhizosphere and root compartments.

**Figure 6:**
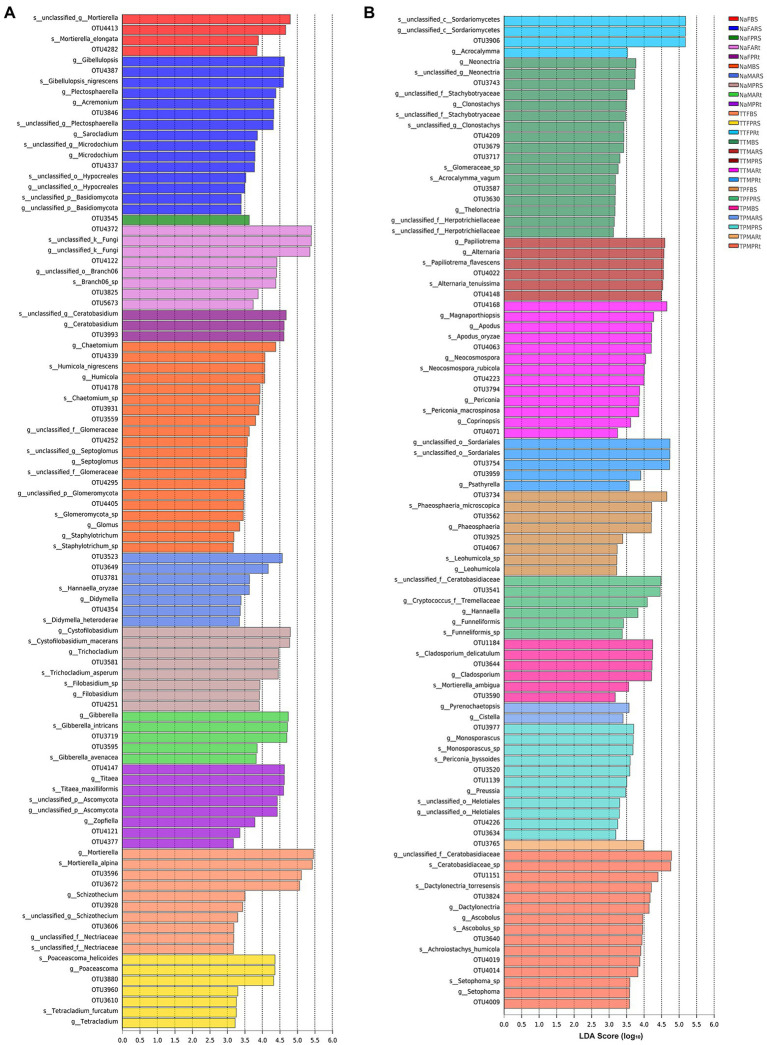
Taxa significantly enriched in bulk soil, rhizosphere and root compartments among 25 sample groups *via* linear discriminant analysis (LDA) by LEfSe. **(A)** Twelve groups in the whole PDF file; **(B)** 13 other groups in the whole PDF file. LDA score (log_10_) = 3; comparison based on all samples-against-all samples from genus to OTU. The treatment details are shown in Figure 1.

In the bulk soil compartment, *Chaetomium*, *Humicola*, *Septoglomus*, *Glomus*, *Staphylotrichum*, and an unclassified genus from the phylum Glomeromycota were significantly enriched in the bulk soil of N778 during maturity (NaMBS; Figure 6), while only *Mortierella* was considerably enriched in the bulk soil of sweet sorghum N778 at the blooming stage (NaFBS; Figure 6A).

In the rhizosphere compartment, *Gibellulopsis*, *Plectosphaerella*, *Acremonium*, *Sarocladium*, *Microdochium*, an unclassified genus of the order Hypocreales, and an unclassified genus of the phylum Basidiomycota were considerably enriched in the rhizospheric soil of N778 aerial roots at the blooming stage (NaFARS; Figure 6A), while only one taxon, OTU3545, as a representative of a completely unclassified genus, was considerably enriched in the rhizospheric soil of N778 primary roots at the blooming stage (NaFPRS; Figure 6A; Supplementary Table S3).

Moreover, OTU3523, OTU3649, OTU3781, and OTU4354, representing the genera *Mortierella*, *Cystofilobasidium*, *Hannaella*, and *Didymella*, respectively, were considerably enriched in the rhizospheric soil of N778 aerial roots at the maturity stage (NaMARS; Figure 6A; Supplementary Table S3). *Cystofilobasidium*, *Trichocladium*, and *Filobasidium* were considerably enriched in the rhizospheric soil of N778 primary roots at the maturity stage (NaMPRS; Figure 6A).

In the root compartment, OTU4372, as a representative of a completely unclassified genus, OTU3825, as a representative of *Poaceascoma*, and an unclassified genus of the order Branch06, including OTU4122 and OTU5673, were significantly enriched in N778 aerial roots at the blooming stage (NaFARt; Figure 6A; Supplementary Table S3), while only OTU3993, which represented the genus *Ceratobasidium*, was significantly enriched in N778 primary roots at the blooming stage (NaFPRt; Figure 6A; Supplementary Table S3). *Titaea*, *Zopfiella*, an unclassified genus of the phylum Ascomycota, and an unclassified genus including OTU4121 and OTU4377, which belongs to the class Sordariomycetes, were significantly enriched in N778 primary roots at the maturity stage (NaMPRt; Figure 6A; Supplementary Table S3), although only the genus *Gibberella* containing OTU3719 and OTU3595 was significantly enriched in N778 aerial roots at the maturity stage (NaMARt; Figure 6A; Supplementary Table S3).

**Figure 7:**
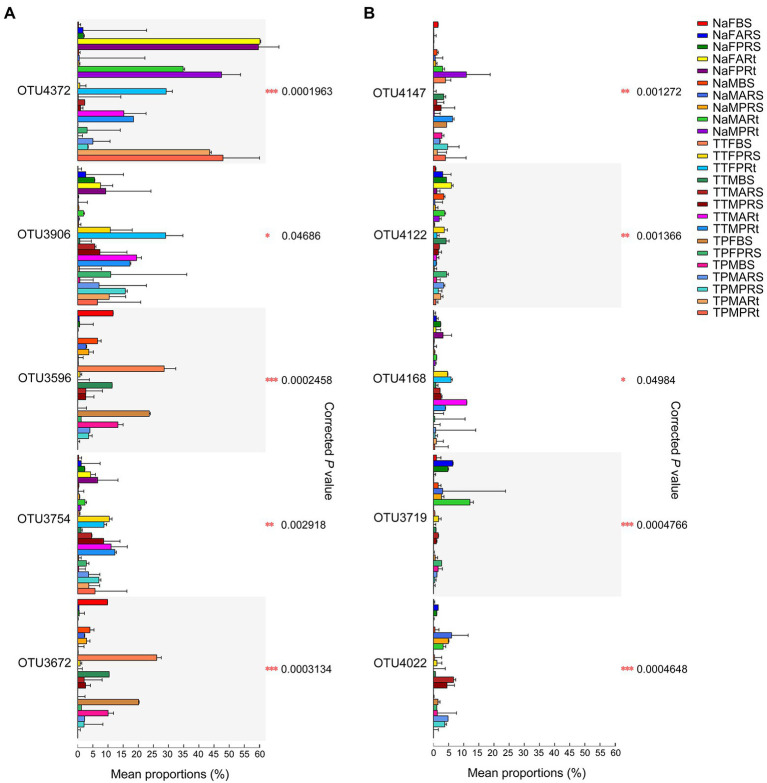
Top 10 OTUs among 25 group comparisons *via* Kruskal–Wallis H test followed by Tukey–Kramer *post hoc* test. **(A)** Top 5 OTUs. **(B)** Top 6th–10th OTUs. The mean proportion (%) is the average relative abundance of taxa in different groups, and the standard deviation (SD) bar is displayed. *, ** and *** indicate *p* < 0.05, *p* < 0.01 and *p* < 0.001, respectively. The treatment details are shown in Figure 1.

Furthermore, 25 group comparisons were carried out by the Kruskal–Wallis H test. At the blooming stage, the proportions of OTU4372 were remarkably higher in N778 primary and aerial root samples than in all bulk soil or rhizosphere soil groups (Figure 7A). Additionally, the proportions of OTU4372 were also considerably or remarkably higher in N778, TP60 and TP213 root samples at the maturity or blooming stage than in all bulk soil or rhizosphere soil groups (Figure 7A). The aforementioned results and LEfSe analysis results indicated that OTU4372 was enriched in sorghum roots, especially in N778 aerial roots at the blooming stage. In addition to OTU4372, the proportion of OTU4122 was considerably higher in NaFARt than in all bulk soil groups (Figure 7B), which was consistent with the result that OTU4122 was significantly enriched in NaFARt by LEfSe analysis (Figure 6A).

However, the proportions of OTU3596 and OTU3672 were remarkably or considerably higher in the bulk soil of TP213 at the blooming stage (TTFBS) as well as the bulk soil of TP60 and N778 at the blooming or maturity stage than in all rhizosphere soil and root samples (Figure 7A). Additionally, LEfSe also revealed that OTU3596 and OTU3672 were significantly enriched in TTFBS (Figure 6A). A similar situation was found in OTU3734 (Supplementary Figure S6), which was significantly enriched in TPFBS *via* LEfSe analysis (Figure 6B). These results indicated that OTU3596, OTU3672, and OTU3734 were depleted in the sorghum rhizosphere and roots, especially in sorghum roots.

### Fungal potential function inferred by FUNGuild

**Figure 8:**
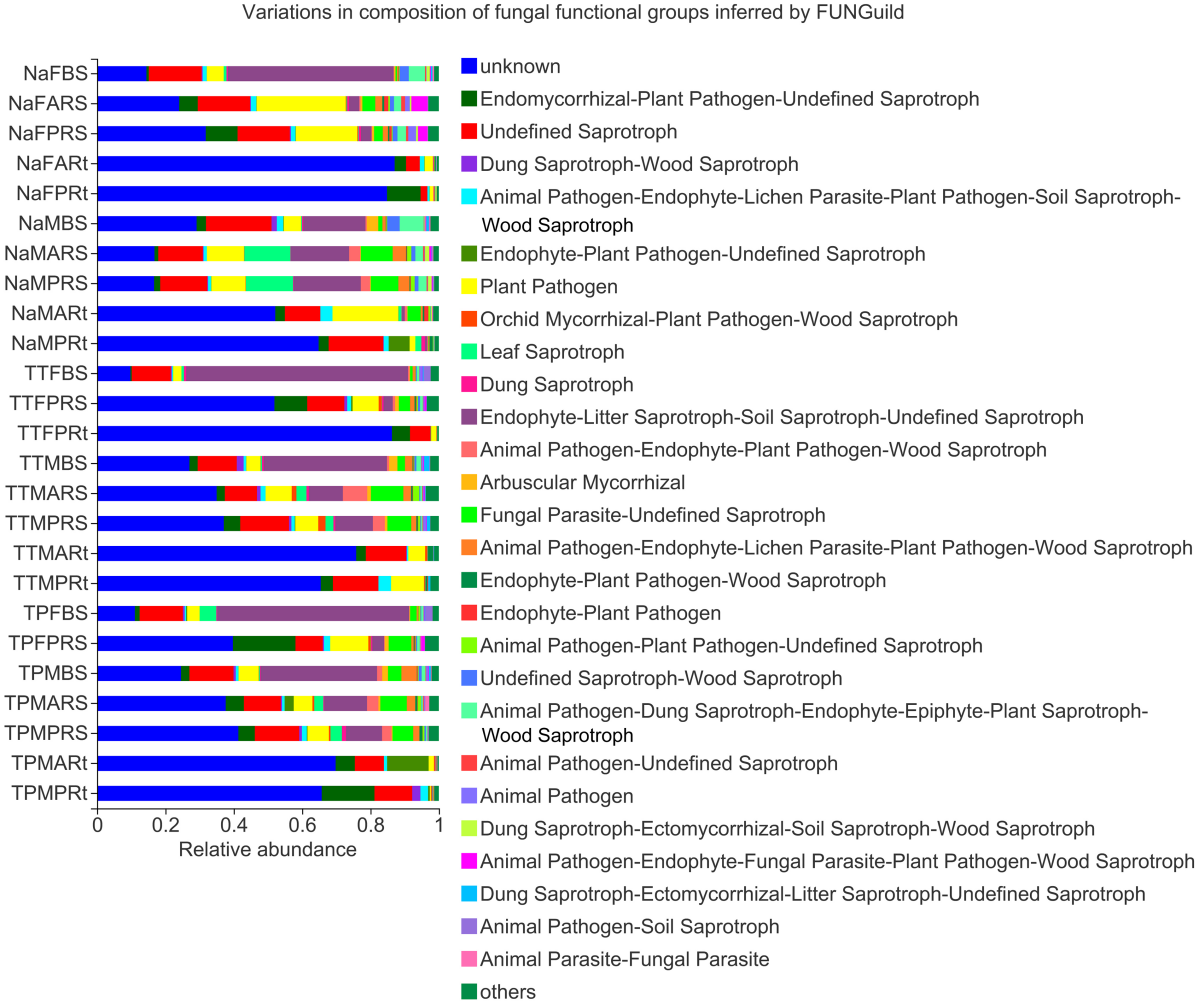
Composition of fungal functional groups inferred by FUNGuild. The treatment details are shown in Figure 1.

To understand the major fungal function of microbiota in different groups of three sorghum lines, the fungal potential function value of each OTU was generated by FUNGuild (Supplementary Table S6), and a composition barplot of different groups was made in the online Marjorbio Cloud platform. At the blooming stage, major fungal potential functions of bulk soil groups, i.e., NaFBS, TTFBS and TPFBS, were endophyte-litter saprotroph-soil saprotroph-undefined saprotrophs, unknown functions, undefined saprotrophs, plant pathogens, leaf saprotrophs, etc. (Figure 8). In addition to the major potential functions, endomycorrhizal or plant pathogen-undefined saprotrophs were obviously enriched in the rhizosphere soil and root samples (Figure 8). Moreover, the unknown function of OTU4372 was significantly or very significantly enriched in rhizosphere soil and root samples (Figure 8).

At the maturity stage, the major fungal potential functions of bulk soil groups, i.e., NaMBS, TTMBS and TPMBS, were similar to those at the blooming stage but accumulated with endomycorrhizal or plant pathogen-undefined saprotrophs, arbuscular mycorrhizal fungi (AMF), fungal parasite-undefined saprotrophs, etc. (Figure 8). Rhizosphere soils of N778 aerial and primary roots were enriched in the functions of leaf saprotrophs, endophyte-litter saprotroph-soil saprotroph-undefined saprotrophs, fungal parasite-undefined saprotrophs, etc., but depleted with animal pathogen-endophyte-fungal parasite-plant pathogen-wood saprotrophs compared with rhizosphere soil at the blooming stage (Figure 8). Additionally, an unknown function of OTU4372 was still major in maturity root groups, although it decreased compared to those at the blooming stage (Figure 8). Also, at the maturity stage, the aerial roots of N778 had many more plant pathogens (Figure 8).

## Discussion

### It makes sense to collect aerial roots and primary roots of sorghum separately

An overview of root-associated microbiota was proposed in a previous report (Lu et al., 2018). In this report, intact sorghum roots were cut into segments and completely washed in PBS without being surface sterilized; as a result, microbiota in the rhizoplane and the root endosphere (Edwards et al., 2015; Lu et al., 2018) were included in the clean sorghum root segments. Hence, the microbiota of clean root segments have more richness and diversity than those of surface-sterilized roots. Furthermore, the Sobs and PD indices of the fungal microbiota in N778 primary roots at the maturity stage and the Shannon index of the fungal microbiota in the primary roots at the blooming stage were significantly different between N778 and the control lines TP213 and/or TP60 (Figures 1J–L). Additionally, a number of OTUs were found only in aerial roots and primary roots of sweet sorghum N778 at the blooming or maturity stage (Figure 4D). Therefore, it makes sense to collect the aerial roots and primary roots of sorghum separately.

### OTU4372, a completely unclassified fungus, was remarkably enriched in the sorghum root compartment

OTU4372, as the top 1st OTU with unknown function, was significantly enriched in the sorghum root compartment, especially in primary and aerial roots of sweet sorghum N778 at the blooming stage (Figures 6, 7) and was found to be a completely unclassified fungus based on the present RDP classifier (v2.11) in this study. Furthermore, the representative sequence of OTU4372 from the T3acMARS sample was retrieved, and its sequence is as follows:

>OTU4372 T3acMARS

TTTCCGTAGGTGAACCTGCGGAAGGATCATTGTCGT

GACCCTTAAACAAAACAGACCGTGAACATGTCTCTCAT

GTCGTCGAGCTTTGGCTCGGCACAAGGTCCCCTTGCTC

CAACCTGGGGCAGAGGGGCCACAAAAGAACCCACGGC

GCCTTAGGCGTCAAGGAACACTCATGTTGCCTTGCACA

GCGGAGTGGTCGGCATGCCTTCCGCTCCCTGAGCAGCG

ATGATATCTTAATCCACACGACTCTCGGCAACGGATATC

TCGGCTCTC.

Megablast, discontiguous megblast, or BLASTn were then performed using the above sequence as a query, but the sequence can not be identified as any known fungal taxon. However, the trophic mode of OTU4372 should at least be symbiotrophs but not pathotrophs or saprotrophs because it is already remarkably enriched in sorghum primary and aerial roots at the blooming stage (Figure 7). Therefore, it is worth further characterizing the phylogenetic position and function of OTU4372.

### *Cladosporium* and *Alternaria* may also be used as dominant indicators of sorghum yield and/or protein content in addition to *Fusarium*

Recently, *Fusarium* and *Guehomyces*, two fungal genera, were reported to be dominant indicators of sorghum yield and protein content, which were present in rhizosphere soil or bulk soil but not in the root endosphere at the maturity stage (Sun et al., 2021); additionally, *Cladosporium*, *Alternaria*, *Preussia*, *Solicoccozyma*, *Netria*, and *Chaetomidium* were reported as other key drivers of sorghum yield or protein content (Sun et al., 2021).

In this study, the genera *Fusarium*, *Cladosporium*, *Alternaria*, *Preussia*, *Chaetomidium*, and *Solicoccozyma* were also found (Supplementary Table S5, sheet of genus_full). However, *Guehomyces* and *Netria* were not found in this study, which might result from the different soil types and sorghum genotypes. Moreover, the total absolute abundances of *Chaetomidium* and *Solicoccozyma* were only 12 and 13 effective tags, respectively, in this study, which might also result from the different soil types and sorghum genotypes.

Furthermore, *Fusarium*, as a pathogenic fungal genus, was found to be present in not only the rhizosphere and bulk soil but also maturity roots of three sorghum lines with high or medium abundance in this study (Supplementary Figure S7A; Supplementary Table S5, sheet of genus_full, highlighted by yellow), which might result from the different methods of sampling roots and/or different soil types. The dominant species of the genus *Fusarium* was *Fusarium commune* in this study (Supplementary Figure S7B).

Additionally, the genus *Cladosporium* was found to exist in not only rhizosphere and bulk soil with high abundance but also the primary and aerial roots of N778 at the maturity stage (NaMPRt and NaMARt) with some relative abundance (Supplementary Figure S7A), although only tiny tags of *Cladosporium* were present in maturity roots of sorghum TP213 and TP60 in this study (Supplementary Figure S7A; Supplementary Table S5, sheet of genus_full, highlighted by yellow). *Cladosporium delicatulum* was the dominant species of *Cladosporium* in this study (Supplementary Figure S7B), and its possible function might be as an endophyte, lichen parasite, plant pathogen, or wood saprotroph (Supplementary Table S6).

The genus *Alternaria* had a similar situation as *Cladosporium*, while its abundance was considerably higher in the rhizospheric soil of N778, TP213, and TP60 at the maturity stage than in the other groups except for the bulk soil of TP60 at the maturity stage (Supplementary Figure S7A; Supplementary Table S5, sheet of genus_full, highlighted by yellow). *Alternaria tenuissima* was the dominant species of the genus *Alternaria* in this study (Supplementary Figure S7B), and its function might be as a plant pathogen or wood saprotroph with soft rot traits (Supplementary Table S6).

Therefore, we hypothesize that the distributions of *Cladosporium* and *Alternaria*, as indicators of sorghum yield and protein content, not only in rhizosphere and bulk soil of three sorghum lines but also in the N778 root compartment with medium abundances at the maturity stage, most likely result from genotypic differences between sweet sorghum N778 and two control sorghum lines. Hence, we propose that, along with *Fusarium*, *Cladosporium* and *Alternaria* in the rhizospheric soil may also be important indicators of sorghum yield and protein content at the maturity stage.

### No dominant fungal taxa with psychrotolerant phenotypes were enriched in the rhizosphere or root of N778 at the maturity stage

Our recently published study revealed the significant enrichment of two dominant bacterial OTUs belonging to *Pseudomonas* and *Pseudarthrobacter* with a potential psychrotolerant phenotype in the rhizospheric soil of N778 at the maturity stage (Lu et al., 2022). Hence, we wondered whether there were dominant fungal taxa enriched in the rhizosphere or roots of N778 at the maturity stage.

*Mucor penicillium* and *Mucor chrysosporium* were identified as psychrotolerant fungi before two decades (Khizhnyak et al., 2003), but those two species were not found in this study. *Sistotrema brinkmannii*, a freezing-tolerant fungus from Antarctic soil, was isolated and characterized in 2010 (Hao et al., 2010), while the abundance of *S. brinkmannii*, i.e., OTU1004 in this study, was very low (Supplementary Table S3). Three years later, two psychrotrophic yeast species, *Rhodotorula mucilaginosa* and *Cystofilobasidium capitatum*, were reported by Sahay et al. (Sahay et al., 2013); however, *R. mucilaginosa*, i.e., OTU2741 in this study, was found but with a very low abundance (Supplementary Table S3); *C. capitatum*, OTU5473, with a low abundance of 443 tags in total, was found in this study, but there was no significant difference between N778 and TP213 or TP60 (Supplementary Table S3).

In 2014, Joshi et al. reviewed the diversity of psychrotolerant phosphate-solubilizing microbes, including *Paecilomyces hepiali* (Joshi et al., 2014). Most of those fungal taxa, including *Paecilomyces hepialid*, *Fomitopsis palustris* and 8 species of the genus *Aspergillus*, were checked, and only two species were found in this study, which were *Fomitopsis palustris* (OTU1313) with very tiny abundance and *Aspergillus terreus* (OTU2193) with a very low abundance of 54 tags in total and mainly present in bulk soil (Supplementary Table S3). Mestre et al. reported three cold-tolerant yeasts, *Aureobasidium pullullans*, *Holtermaniella takashimae* and *Candida maritima*, from Patagonia in Argentina (Mestre et al., 2016); however, three cold-tolerant yeasts were not found in this study.

Recently, approximately 10 yeast strains, including *Saccharomyces cerevisiae*, Mrakia sp., 3 species of *Rhodotorula*, and 4 species of *Naganishia*, were reported as psychrophilic or psychrotolerant fungi (Ganatsios et al., 2019; Tapia-Vazquez et al., 2020; Terpou et al., 2020). Among the above fungi, *Saccharomyces cerevisiae* was not found in this study; 3 species of *Rhodotorula* were found in this study, but all had tiny abundances in total, i.e., 108 tags of *R. ingeniosa* (OTU1186), 72 tags of *R. mucilaginosa* (OTU2741) and 30 tags of *R. paludigena* (OTU4165); 4 species of *Naganishia* were also found in this study but with tiny or very tiny abundances in total, i.e., 95 tags of *Naganishia sp.* (OTU881), 18 tags of an unclassified species (OTU3446), 4 tags of *N. randhawae* (OTU3233) and 3 tags of *N. randhawae* (OTU160).

Gorshkov et al. recently isolated twenty-one harmful fungal strains of *Microdochium nivale* with psychrophilic or psychrotolerant traits from snow mold-affected winter rye and characterized them (Gorshkov et al., 2020). *Microdochium nivale* was not found in this study. However, there were 8 OTUs belonging to the genus *Microdochium*, including OTU4337, OTU4235, OTU263, OTU552, OTU941, OTU949, OTU5178 and OTU983, of which OTU4337 was considerably enriched in the aerial roots of N778 at the blooming stage (NaFARS; Figure 6A), and OTU983 belonged to *Microdochium fisheri*. The effective tag numbers of OTU4337, OTU4235, OTU263, OTU552, OTU941, OTU949, OTU5178, and OTU983 were 3, 959, 664, 48, 12, 19, 2, 1, and 94, respectively (Supplementary Table S3).

Taken together with our recently published work (Lu et al., 2022), our results indicate that N778 can primarily recruit dominant psychrotolerant bacterial taxa but not dominant cold-tolerant fungal taxa into its rhizosphere to support its survival below freezing temperatures.

## Conclusion

This study revealed that almost 4 main alpha diversity indices, Coverage, Sobs, PD, and Shannon, of fungal microbiota in rhizospheric soil and root samples were considerably different between the perennial sweet sorghum cultivar N778 and two control sorghum lines TP213 and TP60 at the blooming or maturity stage. Moreover, PCoA results based on WUF and Bray–Curtis distance metrics revealed that beta diversity in rhizosphere soil of N778 was distinct from those of TP213 and TP60, while beta diversity in root samples of N778 was distinct from those of TP213 but not TP60. Furthermore, LDA and multiple group comparisons revealed that OTU4372, a completely unclassified taxon but with symbiotroph mode, was enriched in sorghum roots, especially in N778 aerial roots at the blooming stage.

Our results indicate that *Cladosporium* and *Alternaria*, two fungal genera in the rhizosphere soil, may also be dominant indicators of sorghum yield and protein content in addition to *Fusarium* at the maturity stage. Our results in this study and published work suggest that the perennial sweet sorghum cultivar N778 can primarily recruit dominant psychrotolerant bacterial taxa, but not dominant cold-tolerant fungal taxa, into its rhizosphere to support its survival below freezing temperatures.

## Data availability statement

The datasets presented in this study are deposited in the CNGBdb, and the CNGB Sequence Archive (CNSA) accession number of raw data from 79 samples is CNP0003362 (https://db.cngb.org/search/?q=CNP0003362).

## Author contributions

G-HL, Z-YN, and XZ conceived and designed the experiments. G-HL, RC, and ZN performed the experiments. G-HL, KZ, and YW analyzed the data. YY, BS, and HY provided resources. G-HL and AF wrote the manuscript. XZ revised the manuscript. All authors contributed to the article and approved the submitted version.

## Funding

This work was supported by the Xiangyu Talent research launch project (31LGH00) and Jiangsu Collaborative Innovation Center of Regional Modern Agriculture and Environment Protection (HSXT3024). The funding sponsors were not involved in the design of the experiments, the research, the interpretation of the data, or the publication of the results.

## Conflict of interest

The authors declare that the research was conducted in the absence of any commercial or financial relationships that could be construed as a potential conflict of interest.

## Publisher’s note

All claims expressed in this article are solely those of the authors and do not necessarily represent those of their affiliated organizations, or those of the publisher, the editors and the reviewers. Any product that may be evaluated in this article, or claim that may be made by its manufacturer, is not guaranteed or endorsed by the publisher.

## Footnotes

^1^ https://db.cngb.org/

^2^ https://db.cngb.org/search/?q=CNP0003362

^3^ http://unite.ut.ee/index.php

^4^ www.majorbio.com

^5^ www.majorbio.com
