# Supplementary material for: Root-associated fungal microbiota of the perennial sweet sorghum cultivar under field growth
Source: Front Microbiol. 2022 Oct 26;13:1026339. doi: 10.3389/fmicb.2022.1026339 (PMC9643593; doi:10.3389/fmicb.2022.1026339)
Supplement: Supplementary file 1 [file Table_1.DOC]

**Table S1 Summary of clean tags, effective tags, OTUs of bulk soil, rhizospheric soil and root samples collected from the perennial sweet sorghum N778 and the control line TP60, TP213 via ITS1 amplicon deep sequencing**

| Sample name | Clean tags  (1737F_2043R) | Mean length of clean tag  (nt) | Q30 of clean tags (%) | Effective taxonomic tags at 97% similarity | OTU number  Before normalization | OTU number after normalization |
| --- | --- | --- | --- | --- | --- | --- |
| Na1FBS | 73476 | 229.80 | 99.2402 | 72710 | 1119 | 977 |
| Na2FBS | 73850 | 229.05 | 99.3939 | 73152 | 1083 | 934 |
| Na3FBS | 70280 | 229.19 | 99.4032 | 68426 | 1030 | 911 |
| Na1FARS | 73344 | 225.32 | 99.0758 | 71554 | 1484 | 1250 |
| Na2FARS | 68395 | 229.08 | 99.1506 | 66882 | 1493 | 1308 |
| Na3FARS | 74240 | 225.73 | 99.2614 | 72276 | 1378 | 1187 |
| Na1FPRS | 72935 | 227.71 | 99.1304 | 71366 | 1543 | 1343 |
| Na2FPRS | 71224 | 232.11 | 99.0829 | 69795 | 1495 | 1304 |
| Na3FPRS | 64769 | 234.19 | 99.0510 | 63028 | 1313 | 1154 |
| Na1FARt | 50319 | 267.33 | **98.3487** | 50199 | 226 | 216 |
| Na2FARt | 47600 | 247.27 | 98.7891 | 47236 | 326 | 319 |
| Na3FARt | 47564 | 255.63 | 98.7551 | 47404 | 247 | 240 |
| Na1FPRt | 60063 | 252.70 | 98.4203 | 59840 | 211 | 187 |
| Na2FPRt | 69593 | 252.72 | 98.7815 | 69396 | 217 | 189 |
| Na3FPRt | 56962 | 258.41 | 98.5307 | 56794 | 217 | 202 |
| Na1MBS | 59603 | 238.10 | 98.9919 | 58778 | 1226 | 1157 |
| Na2MBS | 61548 | 244.04 | 98.6872 | 60963 | 1284 | 1219 |
| Na3MBS | 61075 | 239.73 | 98.8826 | 60092 | 1192 | 1128 |
| Na1MARS | 72706 | 216.41 | 99.5153 | 71676 | 1061 | 882 |
| Na2MARS | 70501 | 225.92 | 99.2549 | 69275 | 1249 | 1057 |
| Na3MARS | 74341 | 221.96 | 99.4962 | 73150 | 914 | 764 |
| Na1MPRS | 71094 | 217.26 | 99.4925 | 70252 | 983 | 825 |
| Na2MPRS | 70832 | 225.49 | 99.2310 | 69722 | 1296 | 1122 |
| Na3MPRS | 69243 | 220.02 | **99.5860** | 68230 | 853 | 729 |
| Na1MARt | 73523 | 249.54 | 98.7136 | 73093 | 405 | 347 |
| Na2MARt | 73879 | 253.59 | 98.6630 | 73559 | 349 | 305 |
| Na3MARt | 72013 | **213.78** | 99.4231 | 71264 | 280 | 231 |
| Na1MPRt | 71661 | 239.74 | 98.8274 | 71285 | 279 | 252 |
| Na2MPRt | 69745 | 264.38 | 98.6815 | 69389 | 313 | 269 |
| Na3MPRt | 66505 | 249.99 | 98.5758 | 66219 | 287 | 253 |
| T1FBS | 56342 | 236.88 | 99.4145 | 56204 | 636 | 608 |
| T2FBS | 66200 | 236.87 | 99.4643 | 66018 | 645 | 607 |
| T3FBS | 70668 | 233.16 | 99.4948 | 70389 | 725 | 673 |
| T1FPRS | 69109 | 225.92 | 99.1316 | 68372 | 1149 | 1014 |
| T2FPRS | 72689 | 231.47 | 99.0479 | 71856 | 1296 | 1150 |
| T3FPRS | 71136 | 230.83 | 99.0549 | 70187 | 1281 | 1123 |
| T1FPRt | 68980 | 237.32 | 98.9385 | 68605 | 203 | 175 |
| T2FPRt | 52761 | 239.88 | 99.1835 | 52616 | 219 | 198 |
| T3FPRt | 72749 | 244.70 | 99.0232 | 72149 | 225 | 188 |
| T1MBS | 49690 | 240.18 | 98.8999 | 49503 | 759 | 752 |
| T2abMBS | 67505 | 234.22 | 99.0904 | 67297 | 951 | 901 |
| T3MBS | 63888 | 237.97 | 99.0737 | 62944 | 1030 | 974 |
| T1MARS | 69737 | 223.21 | 99.1706 | 68962 | 878 | 754 |
| T2deMARS | 56474 | 228.65 | 99.3951 | 56409 | 597 | 567 |
| T3acMARS | 74808 | 230.28 | 99.1280 | 73897 | 1166 | 1003 |
| T3bdMARS | 74174 | 222.77 | 99.1925 | 73030 | 1418 | 1223 |
| T1MPRS | 70051 | 223.56 | 99.4006 | 69602 | 700 | 613 |
| T2deMPRS | 70809 | 221.48 | 99.4314 | 69952 | 972 | 811 |
| T3acMPRS | 73995 | 229.02 | 99.1292 | 73111 | 1139 | 987 |
| T3bdMPRS | 74059 | 229.53 | 99.2015 | 73246 | 1345 | 1141 |
| T1MARt | 69246 | 219.60 | 99.2187 | 68772 | 183 | 162 |
| T2deMARt | 74071 | 240.15 | 98.8134 | 73601 | 225 | 188 |
| T3acMARt | 62789 | 231.74 | 99.2818 | 62705 | 195 | 183 |
| T3bdMARt | 59971 | 238.94 | 99.0313 | 59708 | 161 | 137 |
| T1MPRt | 71662 | 219.99 | 99.4341 | 70509 | 204 | 168 |
| T2deMPRt | 72551 | 240.89 | 99.1777 | 72193 | 167 | 145 |
| T3acMPRt | 59672 | 229.44 | 99.0325 | 59274 | 185 | 168 |
| T3bdMPRt | 51194 | 255.41 | 98.9255 | 51038 | 180 | 171 |
| TP_1FBS | 66004 | 237.11 | 99.3412 | 65839 | 707 | 672 |
| TP_2FBS | 74321 | 228.43 | 99.4969 | 74031 | 760 | 669 |
| TP_3FBS | 65677 | 234.25 | 99.5713 | 65383 | 680 | 613 |
| TP_1FPRS | 73440 | 236.86 | 99.0483 | 72819 | 1256 | 1122 |
| TP_2FPRS | 72824 | 231.50 | 99.1696 | 72041 | 1138 | 997 |
| TP_3FPRS | 53628 | 239.59 | 98.8683 | 53031 | 1142 | 1083 |
| TP_1MBS | 74122 | 237.15 | 99.0826 | 73516 | 1155 | 1013 |
| TP_2MBS | 60991 | 231.83 | 99.3288 | 60650 | 958 | 895 |
| TP_3MBS | 74951 | 232.40 | 98.9256 | 74474 | 1131 | 1011 |
| TP_1MARS | 68345 | 219.77 | 99.1826 | 67818 | 935 | 839 |
| TP_2MARS | 71587 | 238.84 | 99.0882 | 71045 | 852 | 734 |
| TP_3MARS | 72011 | 227.65 | 99.2755 | 71401 | 934 | 828 |
| TP_1MPRS | 74493 | 217.83 | 99.4490 | 74017 | 748 | 633 |
| TP_2MPRS | 71401 | 234.16 | 99.2299 | 71253 | 573 | 537 |
| TP_3MPRS | 66783 | 231.33 | 99.2812 | 66314 | 847 | 759 |
| TP_1MARt | 58886 | 248.15 | 98.7544 | 58580 | 226 | 215 |
| TP_2MARt | 43678 | **270.92** | 98.6331 | **43529** | 175 | 175 |
| TP_3MARt | 43855 | 259.68 | 98.4338 | 43648 | 186 | 186 |
| TP_1MPRt | 71302 | 251.51 | 98.7803 | 71020 | 233 | 187 |
| TP_2MPRt | 74370 | 252.81 | 98.6451 | 73734 | 186 | 154 |
| TP_3MPRt | 48112 | 249.20 | 98.5704 | 47950 | 228 | 222 |

Note: clean tags were obtained after connected tags were filtered to remove chimeras and singleton sequences. Na, T and TP refer to the perennial sweet sorghum cultivar NaPSB778, control sorghum line TP213 and TP60, respectively. F and M refer to the flowering and maturation stages, respectively. BS, RS, ARt and PRt refer to bulk soil, rhizospheric soil, the latest aerial root and the primary root (taproot) samples, respectively, which were collected from each biological replicate containing 5 sorghum plants of N778 or TP60, TP213 except that T2de, T3ac, and T3bd refer to the d and e plants with heavy borer damaged panicles in the 2^nd^ plot, the a and c plants with light borer damaged panicles in the 3^rd^ plot, and the b, d and e plants with heavy borer damaged panicles in the 3^rd^ plot, respectively.
