# Supplementary material for: Root-associated fungal microbiota of the perennial sweet sorghum cultivar under field growth
Source: Front Microbiol. 2022 Oct 26;13:1026339. doi: 10.3389/fmicb.2022.1026339 (PMC9643593; doi:10.3389/fmicb.2022.1026339)
Supplement: Supplementary file 7 [file Data_Sheet_1.docx]

**Root-associated fungal microbiota of the perennial sweet sorghum cultivar under field growth**

**Gui-Hua Lu** ^1, 2^*, Kezhi Zheng ^1^, Rui Cao ^1^, Aliya Fazal ^2^, Zhiye Na ^3^, Yuanyuan Wang ^1^, Yonghua Yang ^2^, Bo Sun ^2^, Hongjun Yang ^3^, Zhong-Yuan Na ^3^*, Xiangxiang Zhao ^1^*

^1^ Jiangsu Key Laboratory for Eco-Agricultural Biotechnology around Hongze Lake, School of Life Sciences, Huaiyin Normal University, Huai’an 223300, China.

^2^ State Key Laboratory of Pharmaceutical Biotechnology, School of Life Sciences, Nanjing University, Nanjing 210023, China.

^3^ Yunnan Eco-Agriculture Research Institute, Kunming 650000, China.

* **Corresponding authors:**

**Gui-Hua Lu**: [ghlu@hytc.edu.cn](mailto:yangyh@nju.edu.cn), Tel/Fax: 86-571-83525992.

Zhong-Yuan Na: 49306346@qq.com, Tel: 86-871-68323648; Fax: 86-871-68322277.

Xiangxiang Zhao: [xxzhao2013@163.com](mailto:xxzhao2013@163.com), Tel/Fax: 86-571-83525778.

**ORCID**

Gui-Hua Lu (0000-0002-5922-6528)

**
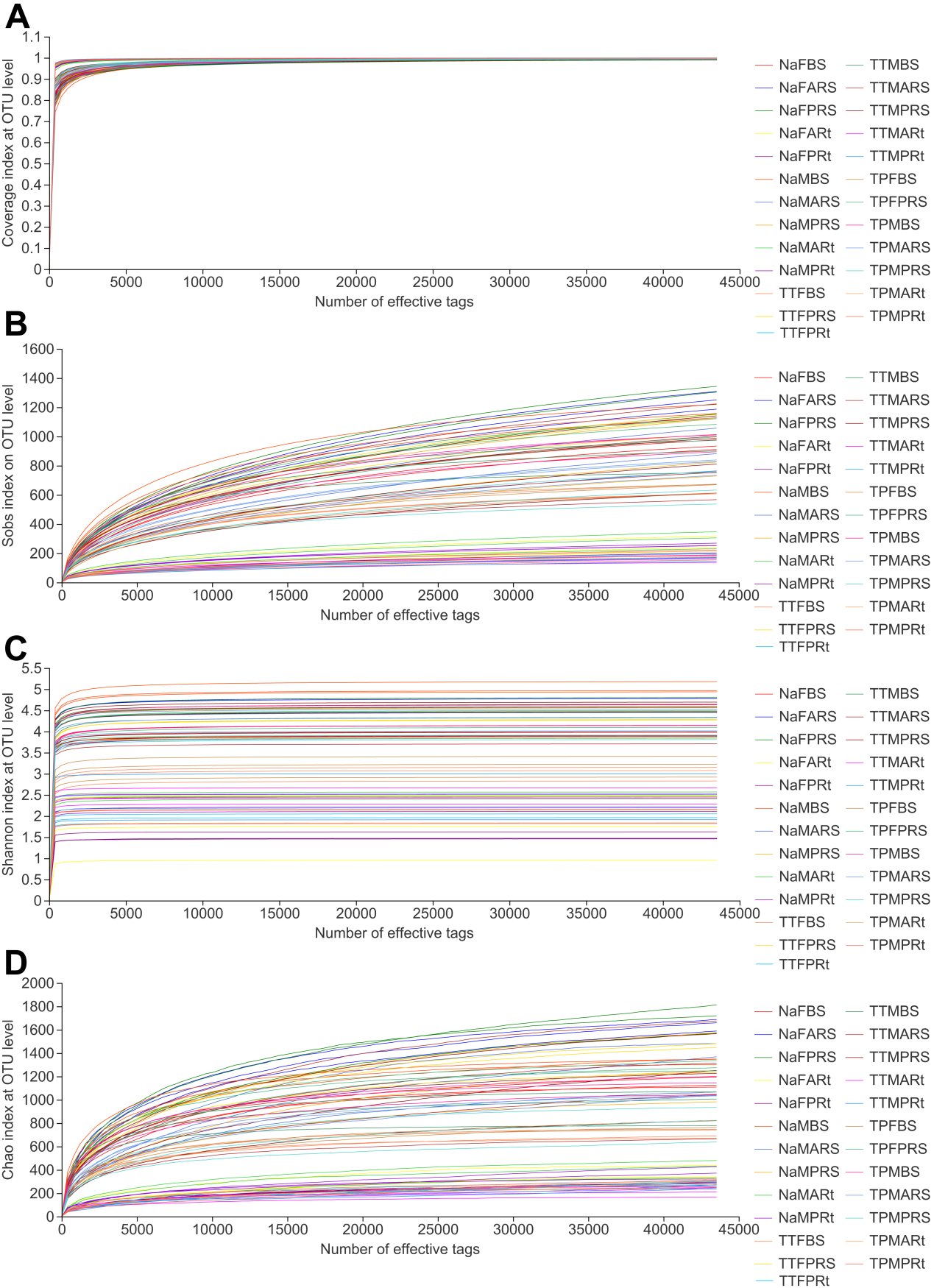
**

**Figure S1. Rarefaction curves of four alpha diversity indices of 25 groups.**

A, B, C, and D represent the Good’s coverage (Coverage), observed OTU richness (Sobs), Shannon, and Chao indices, respectively. For the group name, Na, TT and TP refer to the perennial sweet sorghum cultivar NaPSB778 (N778), the control sorghum lines TP213 and TP60, respectively; F and M represent the blooming stage and the maturity stage, respectively; A and P represent the latest aerial root and the primary roots, respectively; BS, RS and Rt represent bulk soil, rhizosphere soil and root samples, respectively.


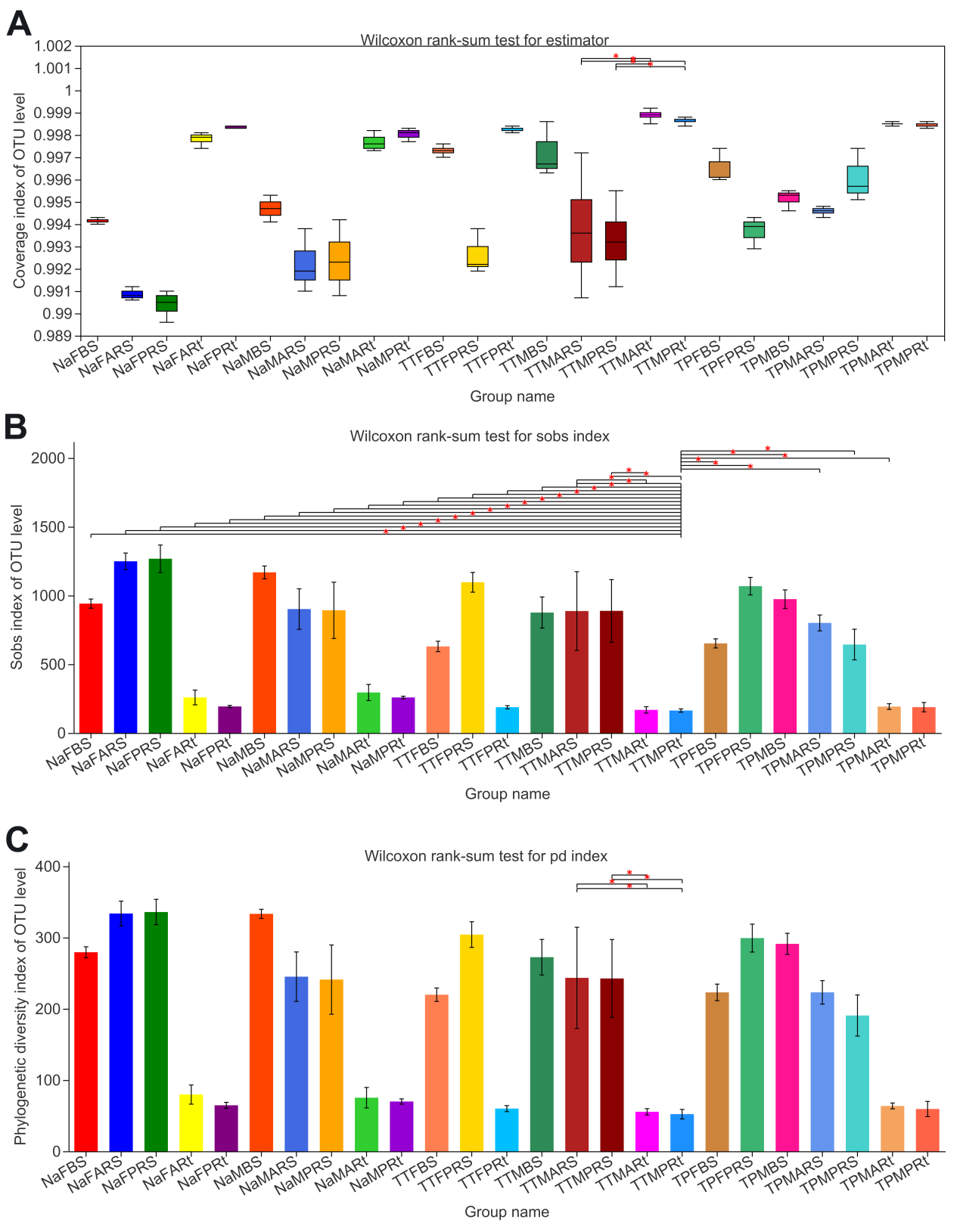


**Figure S2. Differences in alpha diversity among the 25 groups were determined by the Wilcoxon rank-sum t- test.**

(A) Difference in Good’s coverage (Coverage) index. (B) Difference in the observed OTU richness (Sobs) index. (C) Differences in the phylogenetic diversity (PD) index. The treatment details are shown in Figure S1.


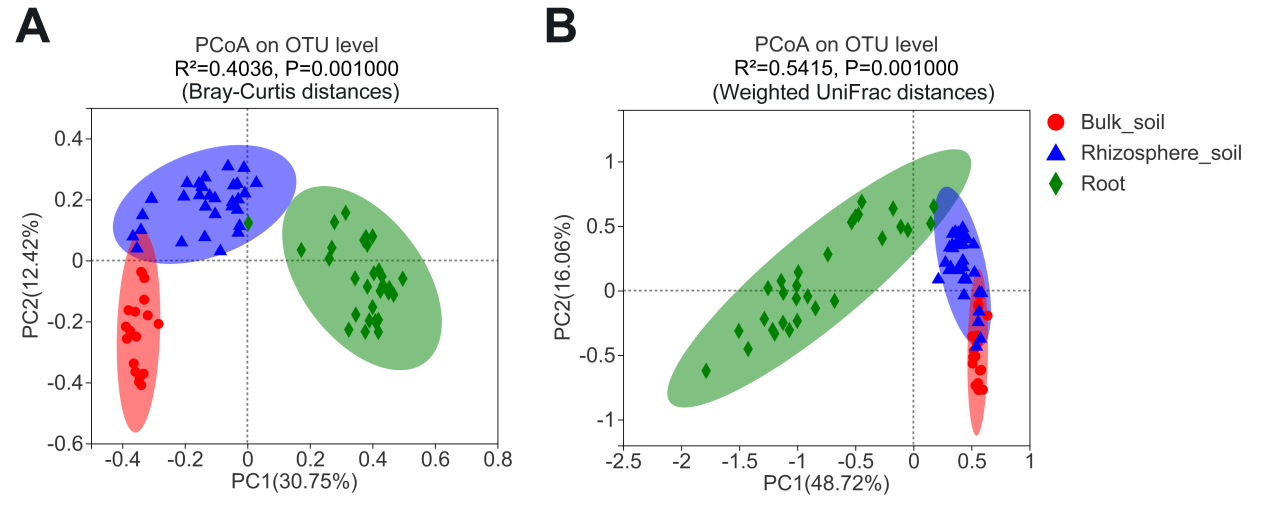


**Figure S3. Beta diversity of fungal microbiota in three scompartments by PCoA.**

(A) PCoA based on Bray–Curtis distance, and the statistical results from Adonis are shown. (B) PCoA based on weighted UniFrac (WUF) distance, and the statistical results from Adonis are shown. The treatment details are shown in Figure S1.


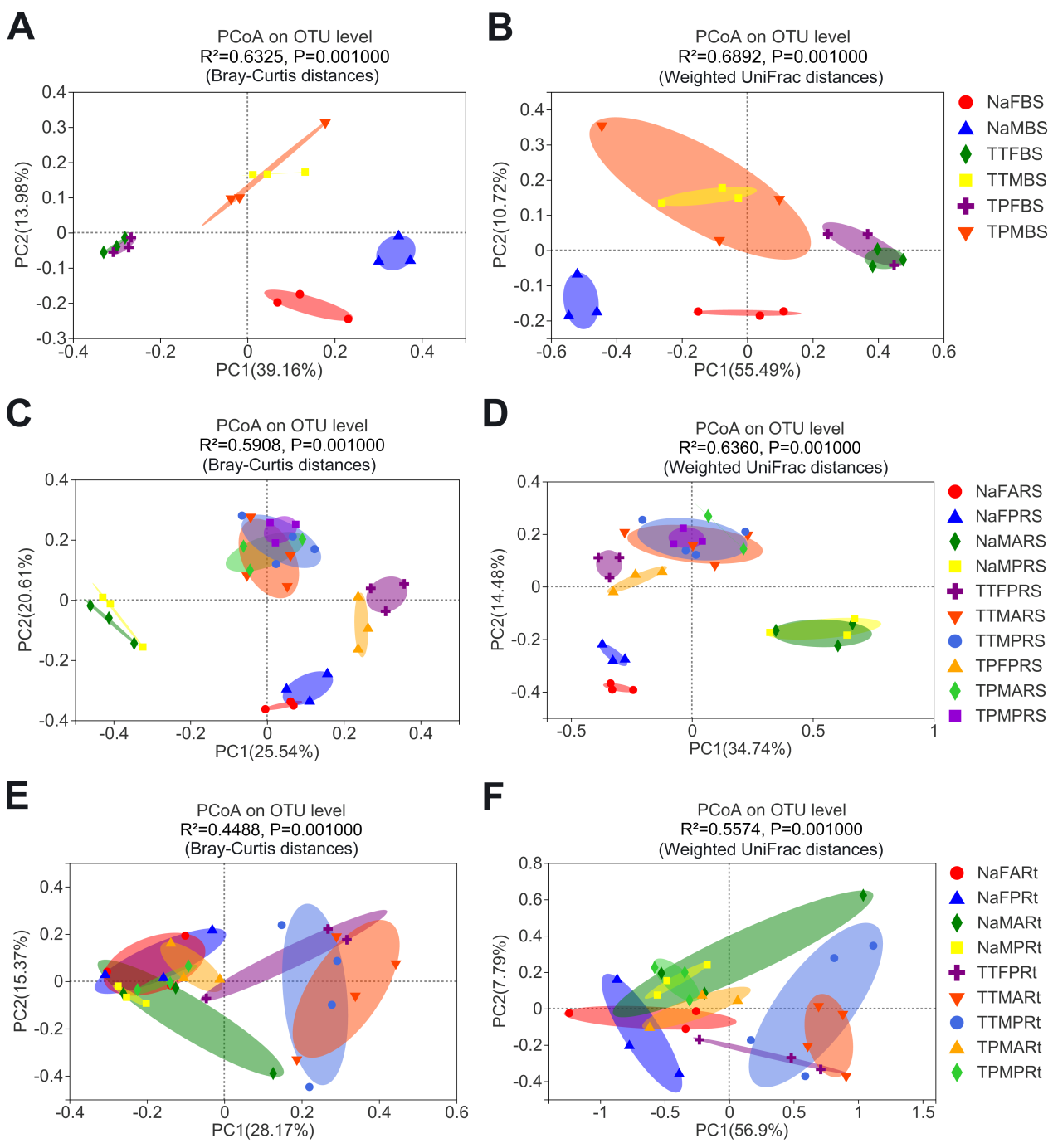


**Figure S4. Beta diversity of fungal microbiota in bulk soil, rhizosphere soil and root compartments, respectively, by PCoA.**

Beta diversity of different groups in bulk soil by PCoA based on Bray–Curtis distance (A) and WUF distance (B). Beta diversity of different groups in rhizosphere soil by PCoA based on Bray–Curtis distance (C) and WUF distance (D). Beta diversity of different groups in roots by PCoA based on Bray–Curtis distance (E) and WUF distance (F). The statistical results from Adonis are shown. and the statistical results from Adonis are shown. The treatment details are shown in Figure S1.


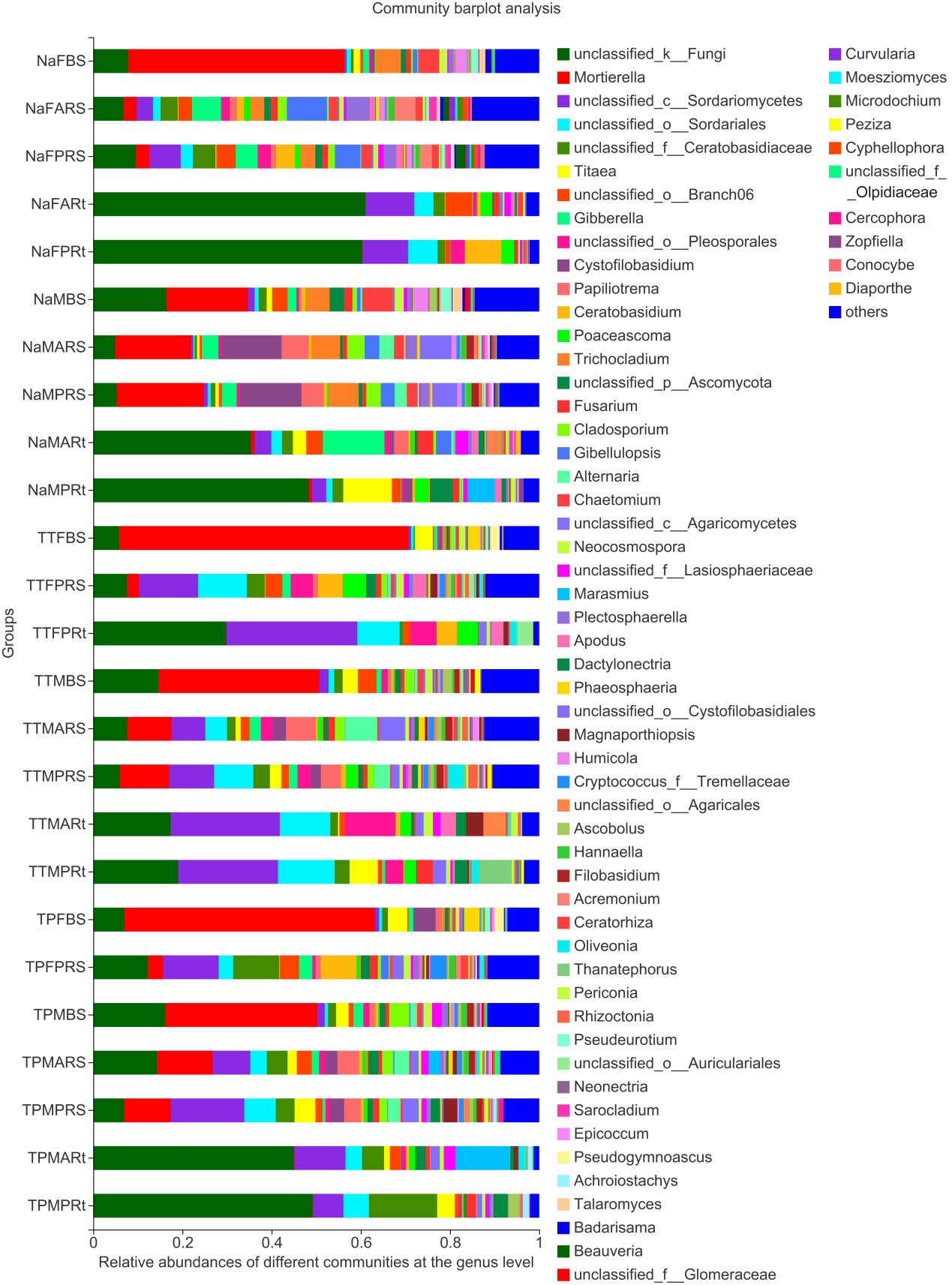


**Figure S5. Fungal microbiota compositions of 25 groups at the genus level.**

Genera with rich abundances are displayed on the stacked column, and those genera with relative abundances less than 1% were combined as others. The treatment details are shown in Figure S1.


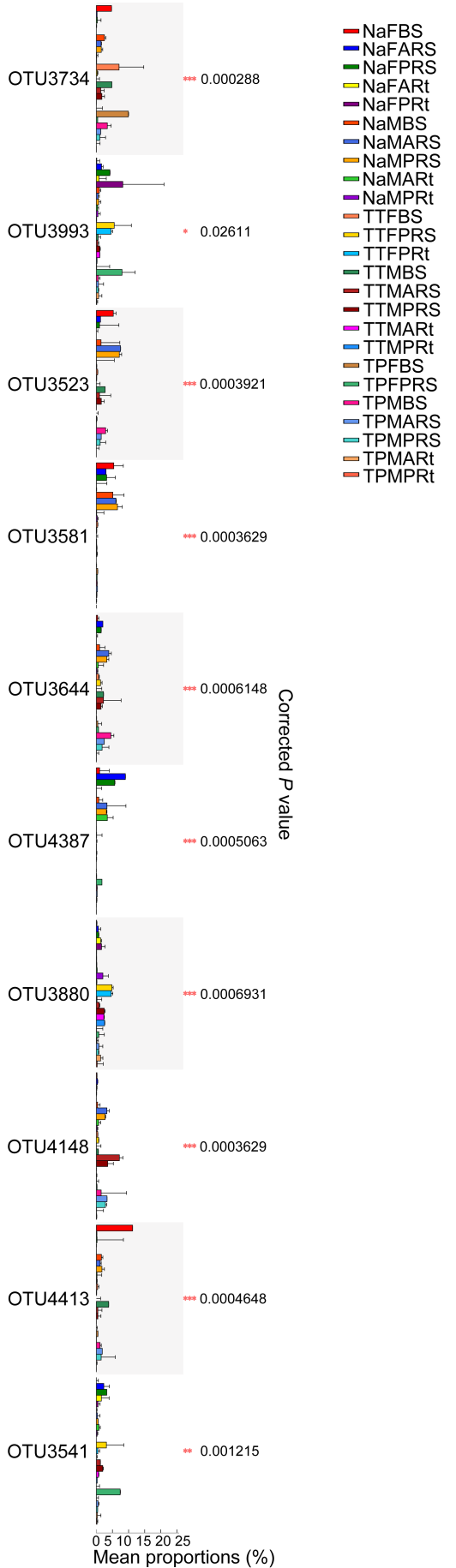


**Figure S6 Top 11^th^ to 20th OTUs among 25 group comparisons via Kruskal–Wallis H test followed by Tukey–Kramer post hoc test.**

The mean proportion (%) is the average relative abundance of taxa in different groups, and the standard deviation (SD) bar is displayed. *, ** and *** indicate *P* < 0.05, *P* < 0.01 and *P* < 0.001, respectively. The treatment details are shown in Figure S1.


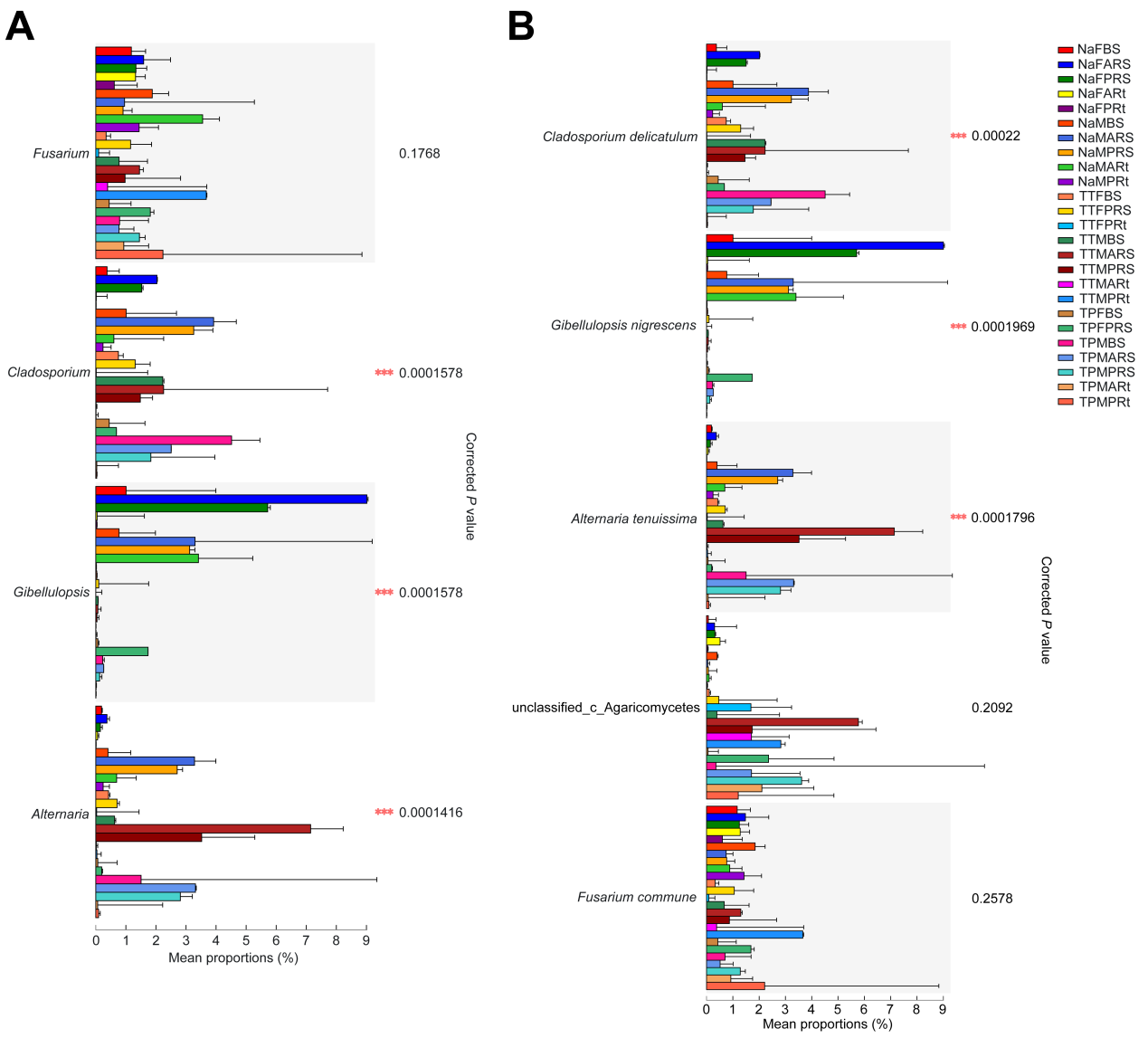


**Figure S7 Three particular genera and their dominant species among 25 group comparisons via Kruskal–Wallis H test followed by Tukey–Kramer post hoc test.**

(A) Top 16^th^ to 19^th^ genera among 25 group comparisons; (B) Top 17^th^ to 21^st^ species among 25 group comparisons; The mean proportion (%) is the average relative abundance of taxa in different groups, and the standard deviation (SD) bar is displayed. *** indicates *P* < 0.001. The treatment details are shown in Figure S1.
